# Bilateral subtrochanteric insufficiency fracture following mini-gastric bypass. A case report

**DOI:** 10.1051/sicotj/2020034

**Published:** 2020-08-28

**Authors:** Jad Mansour, Kaissar Yammine, Anthony El Alam, George Al-Hajj, Chahine Assi

**Affiliations:** 1 Department of Orthopedic Surgery, Lebanese American University Medical Center-Rizk Hospital, Lebanese American University School of Medicine P.O. Box 11-3288 Beirut Lebanon; 2 The Center for Evidence-Based Anatomy, Sports & Orthopedic Research Dubai United Arab Emirates; 3 Department of General Surgery, Lebanese American University Medical Center-Rizk Hospital, Beirut, and Middle East Institute of Health Bsalim Lebanon

**Keywords:** Insufficiency fracture, Mini-bypass bariatric surgery, Vitamin D

## Abstract

Insufficiency fractures are a common complication of bisphosphonate use and have recently been reported in association with Roux-en-Y Gastric Bypass Surgery (RYGB). This study reports a case of a 62-year-old female, 6 years status post Mini Gastric Bypass – One Anastomosis Gastric Bypass (MGB-OAGB), presenting to our institution with bilateral groin pain of 8 months duration unresponsive to conservative management. Diagnostic workup revealed bilateral medial sub-trochanteric insufficiency fractures. She underwent bilateral intramedullary fixation with satisfactory results. This case might suggest a particular fracture pattern in patients undergoing MGB-OAGB, and raises awareness to screen patients with such presentation to rule out a fracture or to prevent the extension of an existing one.

## Introduction

Over the past decade, the prevalence of obesity has nearly doubled and is now considered a global public health concern [1]. When lifestyle modification and pharmacological management fails, bariatric surgery constitutes a suitable long-term treatment modality of morbid obesity [2].

Many complications could occur following bariatric surgery and malabsorption is considered as one of the commonest consequences that could lead to nutritional deficiencies and hormonal disturbances [3]. Among those perturbations, secondary hyperparathyroidism caused by vitamin D deficiency and calcium malabsorption has been reported and may lead to bone loss and an increased risk of secondary and atypical fractures [4, 5].

As for the atypical fractures observed following long-term use of bisphosphonates, the clinical and radiological presentations after obesity surgery are usually similar, particularly in relation to the femoral bone [6]. The increased risk of atypical femur fractures (AFFs) with bisphosphonate use was first described in the last decade [7]. Since, then numerous studies have cited other etiologies affecting bone health and metabolism that correlate with insufficiency fractures [6, 8]. On the other hand, few articles reported the association between bariatric surgery and these fractures. An exhaustive PubMed search reveals that all the reported insufficiency fractures have occurred following a Roux-en-Y Gastric Bypass Surgery (RYGB) [9].

In this paper, we report a case of bilateral medial subtrochanteric insufficiency fracture following a Mini Gastric Bypass – One Anastomosis Gastric Bypass (MGB-OAGB).

Since we are not aware of such an association, a detailed report of this clinical case would be relevant in clinical practice.

## Case report

A 62-year-old female presented to the office complaining of bilateral groin pain that developed 8 months prior to presentation. The patient sought medical consultation at another facility and a radiographic evaluation was conducted revealing negative findings. Symptomatic treatment was prescribed with no improvement. Her past medical history included a homozygote pseudocholinesterase deficiency, a gastric banding 15 years ago, followed by an MGB-OAGB 5 years back. The patient discontinued her prescribed multivitamins 5 years ago. Her body mass index (BMI) at presentation was 25.43 kg/m^2^.

The pain was described as being sharp, intermittent, and radiating to the anterior thigh with no accompanying paresthesia or muscle weakness. It increased in severity with time and activity, especially upon weight bearing and was rated 7/10 using the Visual Analog Scale (VAS).

Physical examination revealed bilateral groin pain on ambulation and tenderness on palpation. Patient had a normal active and passive hip range of motion bilaterally with normal lower extremity neurovascular examination.

**Figure 1:**
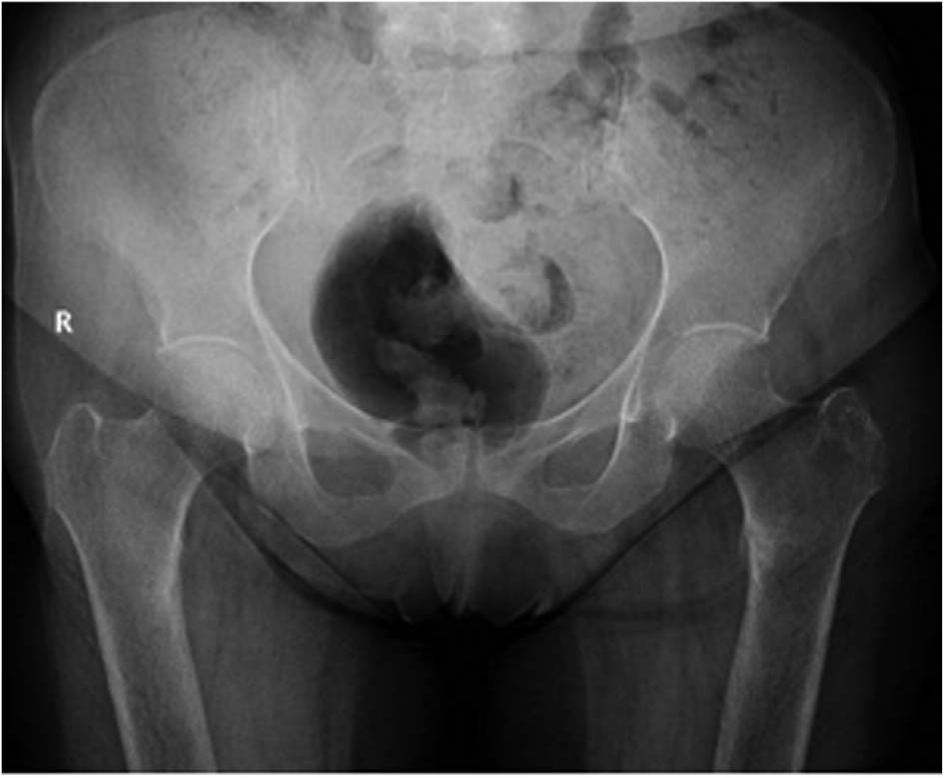
Preoperative antero-posterior pelvis view.

**Figure 2:**
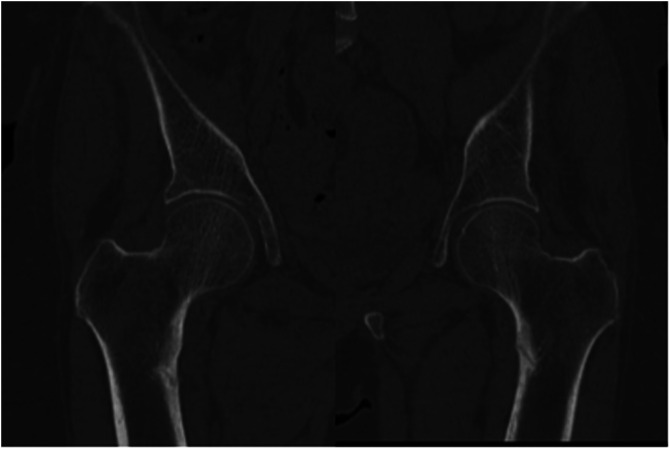
CT scan of both hips.

Pelvic X-rays revealed a bilateral medial unicortical transverse fracture in the subtrochanteric area involving <50% of the femoral canal diameter. A mild periosteal reaction was observed, mainly on the left side, with medial sclerosis surrounding the fracture line (Figure 1). Computed tomography (CT) scan and magnetic resonance imaging (MRI) were done that confirmed the radiological findings of a bilateral subtrochanteric insufficiency fracture involving the medial cortices (Figure 2).

Following clinical and radiological findings, a serum blood test was ordered to rule out bone metabolism abnormalities. Patient was noted to have a vitamin D level of 6 [30–60 ng/mL] and Parathyroid Hormone (PTH) of 395 [15–65 pg/mL]. Sedimentation rate, C-reactive protein, thyroid stimulating hormone (TSH), Calcium, and Phosphorus levels were within the normal range. A parathyroid scintigraphy ordered to rule out a parathyroid adenoma was negative. Findings were consistent with secondary hyperparathyroidism caused by vitamin D deficiency.

**Figure 3:**
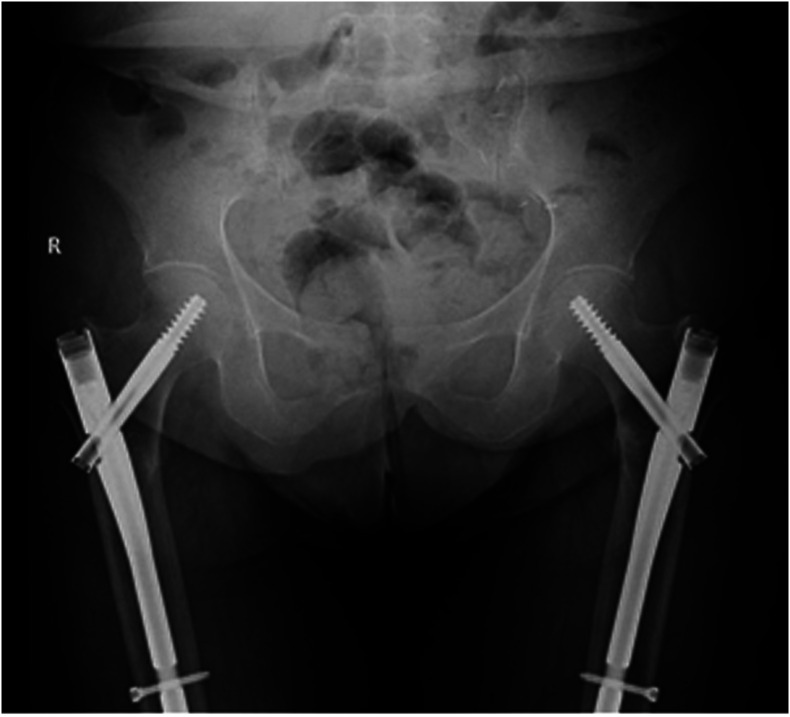
Postoperative antero-posterior pelvis view.

The patient was treated with bilateral prophylactic surgical fixation using short cephalomedullary nails. A full weight bearing postoperative protocol was implemented (Figure 3). Patient was followed-up at 3 weeks, 6 weeks, and 3 months with complete resolution of symptoms.

## Discussion

When lifestyle modifications and pharmacological treatment are ineffective in morbid obesity, bariatric surgery is considered the mainstay of treatment [10]. RYGB is considered the most common bariatric surgery performed in the United States [2]. This procedure is associated with a high rate of malabsorption and change in bone metabolism [11, 12]. Robert et al. demonstrated that the malabsorption risk related to the MGB-OAG procedure is higher than with other techniques [3]. While many studies attempted to explain causes for postoperative bone loss, decreased mechanical weight loading, hormonal changes, and calcium/vitamin D supplementation [13], the pathogenesis of insufficiency fractures following bariatric surgery is still poorly understood in comparison to those following long-term administration of bisphosphonates [8, 14].

A high deficit of calcium and vitamin D absorption is multifactorial; in the MGB-OAG procedure, the duodenum and jejunum where absorption of both calcium and vitamin D occur are bypassed [3]. There are previous reported cases of insufficiency hip fracture all following a RYGB [15]. To our knowledge, the present case is the first reported case of bilateral insufficiency hip fractures following MGB-OAGB surgery. Additionally and while most atypical femoral fracture occur at the femoral neck or below the level of the lesser trochanter, our patient presented with a bilateral insufficiency fracture at the level of the lesser trochanter with medial cortical and periosteal thickening [16]. Though the only abnormalities were a deficit in vitamin D associated with a secondary hyperparathyroidism, the association between a lack of vitamin D and an increased risk of fracture was rarely demonstrated [3]. Mounasamy et al. reported a case of a patient who underwent RYGB but did not comply in taking her vitamin D supplementation, and presented with an incomplete unilateral subtrochanteric femoral fracture [15]. Since these patients have a particular fracture pattern and disturbances of bone and mineral metabolism, a link to vitamin D deficiency should be further investigated [10].

In conclusion, obesity surgery including mini-bypass technique could induce a risk of developing atypical subtrochanteric fractures. Serum level indicators of bone metabolism should be monitored for a long-term follow-up after any gastric bypass surgery. We hope that our case report would help clinicians to bear in mind the differential diagnosis of insufficiency fracture when confronting an atypical, atraumatic hip pain following bariatric surgery.
